# Investigation of NH_3_ Desorption Kinetics on the LTA and SOD Zeolite Membranes

**DOI:** 10.3390/membranes12020147

**Published:** 2022-01-25

**Authors:** Natalya E. Gordina, Tatyana N. Borisova, Ksenya S. Klyagina, Irina A. Astrakhantseva, Alexander A. Ilyin, Ruslan N. Rumyantsev

**Affiliations:** Laboratory of Synthesis, Research and Testing of Catalytic and Adsorption Systems for Hydrocarbon Processing, Ivanovo State University of Chemistry and Technology, Sheremetievskiy Ave., 7, 153000 Ivanovo, Russia; gordinane@mail.ru (N.E.G.); klyagina-2001@mail.ru (K.S.K.); i.astrakhantseva@mail.ru (I.A.A.); ilyin@isuct.ru (A.A.I.); rnr86@ya.ru (R.N.R.)

**Keywords:** zeolite membrane, temperature-programmed desorption, acid-base properties, selectivity, activation energy

## Abstract

The acidity characteristics of zeolite are highly significant, and understanding the acidic properties is essential for developing new types of zeolite catalysts. Zeolite membranes were synthesized using metakaolin, sodium hydroxide, and alumina with a molar ratio of 6Al_2_Si_2_O_7_:12NaOH:2Al_2_O_3_ as the starting ingredients. X-ray diffraction, scanning electron microscopy, and infrared spectroscopy were used for this study. N_2_ adsorption measurements determined the surface areas of the SOD zeolite membrane (115 m^2^/g) and the LTA membrane (150 m^2^/g). The units of absorbed water vapor were 40 and 60 wt% for the SOD membrane and the LTA membrane, respectively. The strength and number of acid sites of the synthesized LTA and SOD zeolite membranes were determined by temperature-programmed desorption of ammonia. As a result, the value of the total acidity of the LTA zeolite membrane is in the range of 0.08 × 10^19^ units/m^2^ while that of the sodalite membrane is an order of magnitude lower and is 0.006 × 10^19^ units/m^2^. The apparent activation energy values for desorption of ammonia from LTA and SOD zeolite membranes were calculated using data on the kinetics of desorption of ammonia at different heating rates. It was found that at temperatures below 250 °C, the degree of conversion of the activation energy values is no more than 35 kJ/mol, which corresponds to the desorption of physically bound ammonia. An increase in the activation values up to 70 kJ/mol (for SOD) and up to 80 kJ/mol (for LTA) is associated with the desorption of chemically bound ammonia from the samples.

## 1. Introduction

Zeolites and membranes based on them are widely used in various separation processes of mixtures and gases in pressure swing adsorption (PSA) processes [1,2]. The use of zeolite membranes in PSA processes is determined by their capacity to separate at the molecular level [3]. The study of the economic feasibility of PSA on zeolites when obtaining oxygen in air separation units showed that the energy intensity of this process is significantly lower than in liquid air rectification units of the same efficiency [4,5]. However, under the conditions of PSA processes, zeolites tend to exhibit variability in capacitance characteristics and a strong dependence of the adsorbent properties on the preparation, storage, and use [6,7,8,9]. Therefore, one of the main indicators is the properties of the surface of the adsorbent, which play an essential role in predicting their use in PSA processes.

The developed acidic properties of zeolites arise due to the inclusion of an aluminum atom in the tetrahedral region of the framework; the acidity depends on the zeolite framework microstructure, including the presence, number, and size of cavities. Zeolite membranes are among the most promising materials used on an industrial scale due to their unique properties, such as a high uniformity and pore size and high thermal and chemical stability. Zeolite membranes are the only membranes capable of performing separation at the molecular level [10,11]. In this case, the study of zeolite membranes is carried out for further prediction and use in low-cycle adsorption processes, and for gas separation and pervaporation. The factors determining the use of zeolite membranes in these processes include the acid-base properties of the surface and the structural features of the assembly of the zeolite framework.

Among the variety of methods used for measuring surface acidity, due to the availability, speed, and good visualization of results, the most popular method is temperature-programmed desorption (TPD) [11,12,13]. The use of TPD makes it possible to record the energy spectrum of samples by desorption of probe substances, and to quantify the concentrations of active centers and the activation energies of desorption of probe substances in various forms of desorption [11,12,13].

Thus, the purpose of this study was to investigate the surface properties of materials based on zeolites and their further use in pressure swing adsorption processes.

## 2. Materials and Methods

Powder X-ray diffraction (XRD) patterns were recorded on a Bruker D8 X-ray diffractometer (Bruker, Billerica, MA, USA). Copper Kα radiation (*λ* = 0.15406 nm, Ni–filter) was used with a 40 kV and 20 mA power supply. The scan rate was 1 min^−1^, and the scanning step was 0.01. The crystalline phases in the XRD patterns were identified by comparison of the calculated interplanar spacings (*d* = *λ*/2 sin *Θ*, where *λ* is the wavelength and *Θ* is the diffraction angle) with those taken from the ASTM database.

Since the LTA and SOD zeolites used in this study have a cubic structure, the zeolite lattice parameters were calculated as:

(1) $$\frac{1}{d^{2}}=\frac{h^{2}+k^{2}+l^{2}}{a^{2}}$$

where *h*, *k*, and *l* are Miller indexes.

Broadening of the X-ray diffraction profile allows one to determine both the dimension of the coherent scattering region (CSR) and the value of mean-square micro deformations (MDs). For this purpose, we used the modified Scherrer’s equation [14,15]. The modified Scherrer’s equation (so-called Scherrer–Selyakov equation) can be written as:

(2) $$\beta_{ph}=\frac{\lambda }{D_{CSR}\cos\Theta }+4\varepsilon \tan\Theta$$

or in a linear form:

(3) $$\beta_{ph}\cos\Theta =\frac{\lambda }{D_{CSR}}+4\varepsilon \tan\Theta$$

where *β_ph_* is the physical component of the broadening, DCSR is the CSR dimension, *ε* is the mean-square MD value, and *Θ* is the position of the profile centroid of the sample. The value of *β_ph_* can be extracted from the total broadening profile using a Gaussian distribution as:

(4) $$\beta_{s}^{2}=\beta_{p}{{}_{h}}^{2}+\beta_{s}{{}_{t}}^{2}$$

where *β_s_* is the integral half-width of the sample profile and *β_st_* is the integrated half-width of the standard sample. It was assumed that for the standard sample (Ecolab, Moscow, Russia), the measured broadening is equal to the instrumental broadening only and is associated with both the device characteristics and exposure conditions.

The Fourier transformed infrared (IR) spectra were measured using an Avatar 360 FT–IR ESP spectrometer (Thermo Fisher Scientific, Waltham, MA, USA) in the range of 4000–400 cm^−1^. The samples were prepared using the KBr method with a sample-to-KBr ratio of 1:100.

In this study, ammonia was used as a probe to investigate the acid-base properties of zeolite structures. The choice of ammonia is due to its high degree of basicity, which makes it possible to determine strongly acidic centers and weak centers by the small size of the given molecule [11,13].

The concentration of acid sites in the investigated samples was identified by the units of ammonia desorbed at the moment of fixation of the desorption peaks. The accuracy of determining the units of desorbed ammonia by gas chromatography was ±5%.

Temperature-programmed desorption of NH_3_ was performed using a Setaram DSC 111 differential scanning calorimeter (Caluire, France) consisting of a flow measurement and switching system and a cylindrical oven controlled by a linear temperature programmer (Omega CN 2010). A mass spectrometer (MS, Thermostar from Pfeiffer, Aßlar, Germany) was used as a detector.

The adsorption of NH_3_ estimated the total surface acidity of the samples by the number of chemisorbed molecules. The heating rate was 10, 15, 20, 25, and 30 °C/min. The following formula determined the number of active centers:

(5) $$N_{i}=\frac{6\cdot 10^{23}{}^{}\cdot S\left(T_{maxi}\right)\cdot V}{22,400\cdot S_{s}{}_{p}\cdot \sum S\left(T_{m}{}_{axj}\right)\cdot m}$$

where 6.022 × 10^23^ is Avogadro’s number; *S*(*T_maxi_*) is the area under the corresponding maximum on the thermal desorption curve, mm^2^; *S_sp_* is the specific surface of the support samples, m^2^/g; *G* is the weight, g; and Σ*S* (*T_maxi_*) is the total area of peaks on the thermal desorption curve, mm^2^; mL. The number of moles of desorbed ammonia was calculated by *V*/22,400, where *V* is the desorbed volume of ammonia (mL, NTD).

Samples for TPD were loaded into a quartz glass tubular reactor, and the resulting layer was fixed with quartz wool. Before taking the TPD values of ammonia or carbon dioxide, the sample was heated to 650 °C for 60 min in a He flow (30 mL/min) to remove adsorbed components, such as H_2_O. The adjusted pretreatment temperature provided reproducible TPD conditions and avoided sintering effects. Then, the sample was cooled to 50 °C. The adsorption of ammonia was carried out from an ammonia-gel mixture with a concentration of 10 vol.% NH_3_. After saturation, it was washed with He.

N_2_ adsorption-desorption isotherms were measured at 77 K on a Sorbi–MS analyzer (Novosibirsk, Russia). Samples were outgassed at 573 K before the adsorption measurements. The specific surface area was calculated from nitrogen adsorption data in the relative pressure range from 0.05 to 0.2 using the BET (Brunauer–Emmett–Teller) equation. The total pore volume was estimated from nitrogen adsorbed at a relative pressure of about 0.99.

The scanning electron microscopy (SEM) measurements were taken with a JSM–6460 LV microscope (JEOL, Ltd., Tokyo, Japan).

To synthesize the zeolite membrane, we used kaolin for the perfume industry (Prosko Resursy, Dnipro, Ukraine). The content of the kaolinite phase was 97.7 wt%. Metakaolin, in the form of an amorphous fine-dispersed white powder, was prepared by the calcination of kaolin at 700 °C for 4 h. Moreover, commercial sodium hydroxide (Kaustik, Volgograd, Russia) in the form of flakes was used. The NaOH content was 99.5 wt%. Commercial aluminum hydroxide (SUAl, Shelekhov, Russia) was used. The aluminum hydroxide contained 98.8 wt% gibbsite. Aluminum hydroxide was converted to γ-Al_2_O_3_ as an amorphous fluffy white powder during the calcination process at 550 °C for 4 h.

To synthesize membranes, amorphous matrices based on porous anodic alumina separated from an aluminum support (10 × 10 nm matrix, 20 nm diameter, 20 μm thickness; manufactured by Nelan-Oxide Plus, Petrozavodsk, Russia) were used as a support.

In the first step, to fix the components on the support surface, the support was immersed in the initial suspension and subjected to USP (ultrasonic-assisted processing) for 10 min. The molar ratio of the mixture ingredients was Al_2_Si_2_O_7_/NaOH/γ-Al_2_O_3_ = 6: 12: 2. This ratio was selected to ensure sodium aluminate formation in the first step of LTA and SOD zeolite membrane synthesis. For this purpose, excess γ-Al_2_O_3_ was additionally introduced into the initial reagent mixture. The optimal γ-Al_2_O_3_ excess is 2 mol alumina per 6 mol metakaolin (33.3 mol%). Suspensions with a mass weight ratio of solid to liquid (S/L) of 5: 1 (with distilled water as a solvent) were prepared from the initial mixture.

Zeolite membranes were synthesized by solution crystallization without seed crystals. The crystallization step utilized a 2 mol/L and 8 mol/L NaOH solution in appropriate units to ensure S/L = 10: 1 (volume ratio). Al_2_O_3_ supports with a pre-applied layer of sodium aluminate/aluminosilicate precursors prepared in the previous USP step were dried at 60–80 °C and calcined at 650 °C before they were immersed in the solution. Crystallization was carried out with two solutions by conventional heating in a 2 and 8 mol/L NaOH solution (pH 14) at 80–90 °C for 2 h, for the synthesis of the LTA and SOD zeolite membrane, respectively.

## 3. Results and Discussion

The main factor determining the selectivity of zeolites and the main distinguishing feature from other materials is the shape and size of the pores and cavities in their structure. Considering membranes based on LTA and SOD zeolites, the optimal parameters for their production were defined in [8,9].

**Figure 1:**
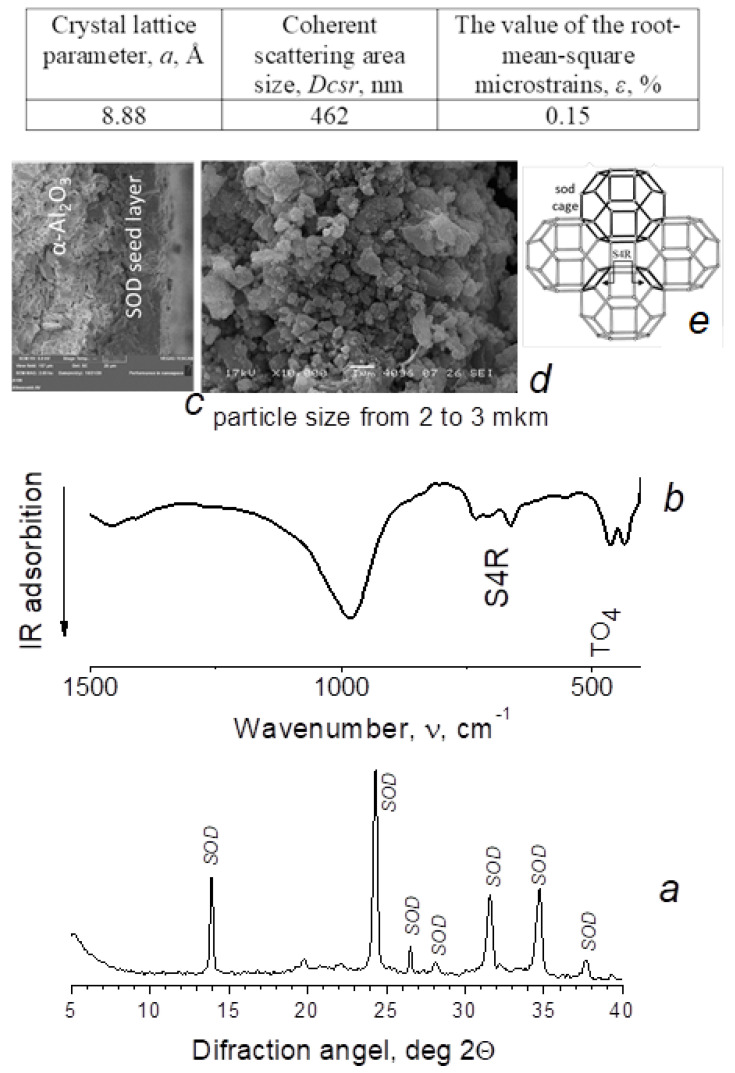
X-ray diffraction pattern (CuKα radiation) (**a**), IR spectrum (**b**), SEM image (**c**) cross-section, (**d**) top view), and (**e**) SOD zeolite membranes structure.

**Figure 2:**
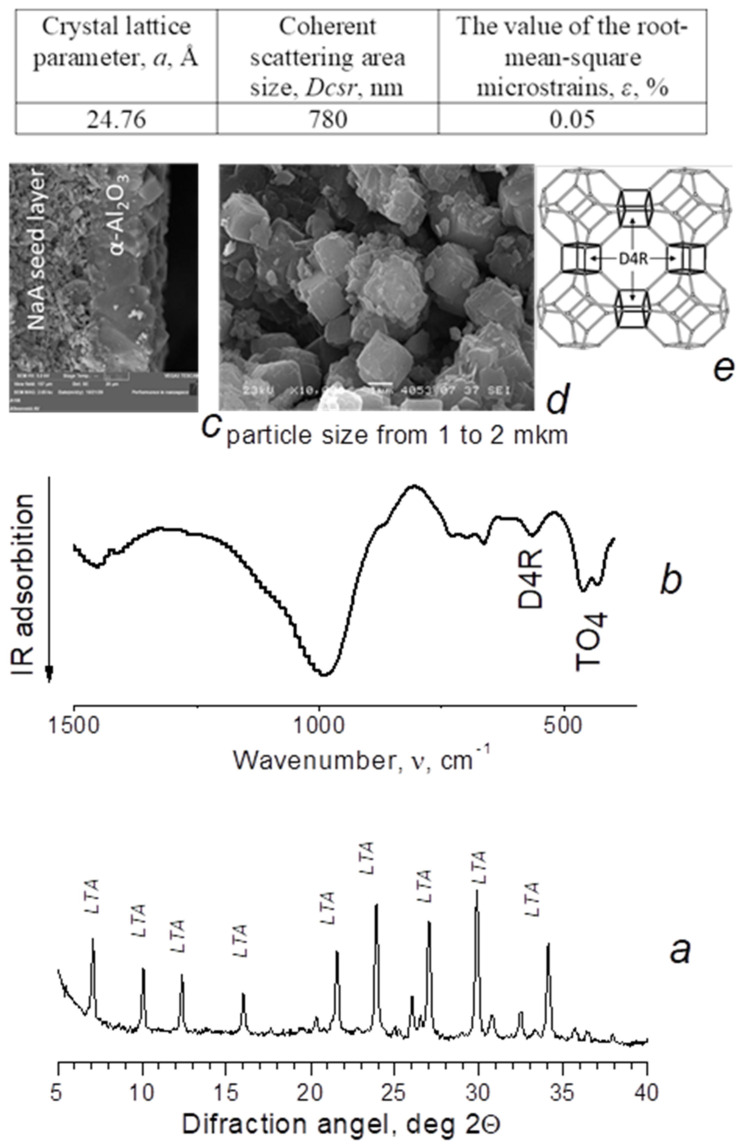
X-ray diffraction pattern (CuKα radiation) (**a**) IR spectrum; (**b**) SEM image; (**c**) cross-section; (**d**) top view; (**e**) LTA zeolite membranes structure.

The main characteristics of the obtained membranes are shown in Figure 1 and Figure 2. We can see that the pore size of the crystal lattice a of the obtained samples is close to theoretical values and corresponds to SOD = 8.88 Å, LTA = 24.76 Å. The crystal lattice deformation level is insignificant and does not exceed 0.15%. The calculated values of the coherent scattering region (Dcsr) are 462 nm for SOD (Table in Figure 1) and 780 nm for LTA (Table in Figure 2), which is natural and takes into account the differences in the arrangement of the sodalite structures of these samples. In the case of sodalite, cuboctahedra are linked through simple four-membered rings (Figure 1e), whereas with LTA zeolite, they are linked through double four-membered rings (Figure 2e). The cuboctahedra link forms a system of regular large α-cavities with a diameter of 1.1 nm (LTA zeolites), connected by rings 0.4–0.5 nm in diameter. Such rings are spaces that open access to the volume in which molecules are adsorbed. Similar to amorphous adsorbents, rings are identified with pores. LTA zeolite is characterized by small micropores 0.3–0.45 nm in size [14].

The total surface acidity of the samples was estimated from the adsorption of NH_3_ and the number of chemisorbed molecules, based on the assumption that each probe molecule occupies one acid-base center on the surface [13,15,16,17,18,19]. At the same time, regardless of the type of membranes, gas desorption was fully completed with the temperature increase in the reactor to 600 °C. In general, if the desorption of physically adsorbed molecules does not require thermal activation, it is heating that contributes to the beginning of the desorption of chemisorbed probe molecules. Desorption takes place from the same centers in a specific temperature range, and, consequently, the typical maximum temperature characterizes the bond strength (acid-base strength of the surface center).

**Figure 3:**
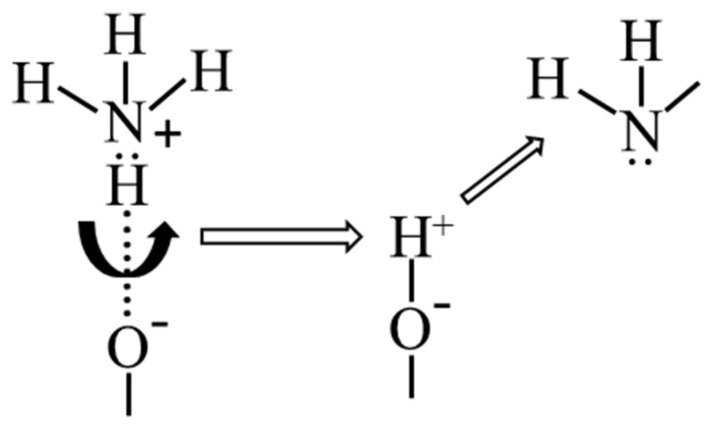
Schematic illustration of desorption of weakly bound ammonia molecules from acid sites upon heating.

When identifying acid sites on the surface of metal-containing samples by the TPD of ammonia, the choice of a temperature of 200 ± 50 °C was proposed as a conventional boundary [13]. Thus, below this value, desorption of ammonia is associated with weak Bronsted centers in the form of OH-NH_3_ adducts (surf). At a higher temperature, molecules held on the surface by stronger (Lewis) acid centers are desorbed. Lewis acid and basic centers are coordinatively unsaturated surface cations and anions, respectively. This phenomenon is characterized by vacancies in the coordination sphere and a strong covalent bond with an NH_3_ molecule [17,18,19]. At the same time, the acid sites in zeolites cannot be explained only by the presence of hydroxyl groups. An investigation into the adsorption of pyridine on zeolite, shown in [20], revealed that after heating the sample with adsorbed pyridine at 200 °C, the formation of pyridinium ions was observed in the spectra, which indicates the presence of Bronsted centers in the zeolite. It is not clear whether these centers are formed during the rearrangement of the zeolite framework and the migration of traces of water in the zeolite under calcination or whether they are formed as a result of decomposition of the adsorbed pyridine. A schematic representation of the process of desorption of ammonia is shown in Figure 3.

**Figure 4:**
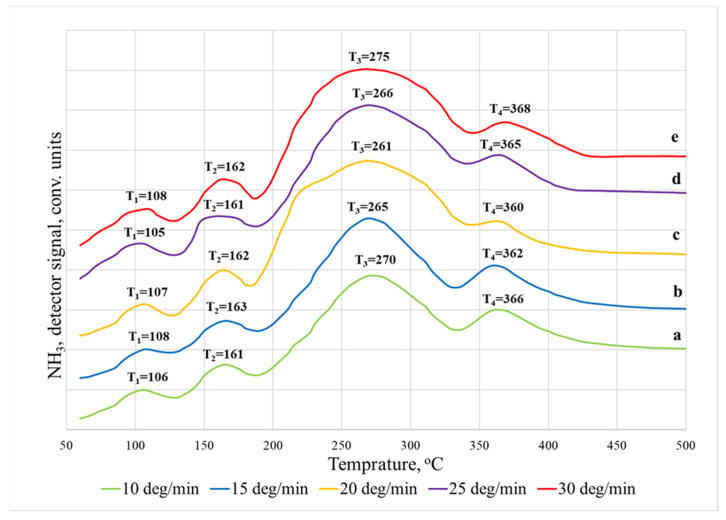
Thermal desorption spectra of NH_3_ on SOD membranes: (**a**) 10 °C/min; (**b**) 15 °C/min; (**c**) 20 °C/min; (**d**) 25 °C/min; (**e**) 30 °C/min.

It was found that the TPD spectra of ammonia desorbed from the surface of the sodalite membrane (Figure 4) exhibit four desorption temperature maxima (T_1_–T_4_). They can be conventionally ranked as weakly acidic (T_1_, T_2_) and moderately acidic (T_3_), and strong (T_4_). In this case, the desorption of ammonia was completely accomplished when the temperature in the reactor increased to 500 °C. From the data of the TPD spectra recorded at different heating rates, it can be clearly seen that the heating rate does not significantly affect the position of the peaks. The low-temperature region up to ~200 °C is characteristic of the desorption of physically adsorbed ammonia molecules adsorbed on unsubstituted cationic sites (100–120 °C), and the desorption of ammonia from weak acid sites (150–170 °C) [18,19,21]. The high-temperature region at 250–500 °C corresponds to the desorption of ammonia adsorbed on medium and strong acid sites.

**Figure 5:**
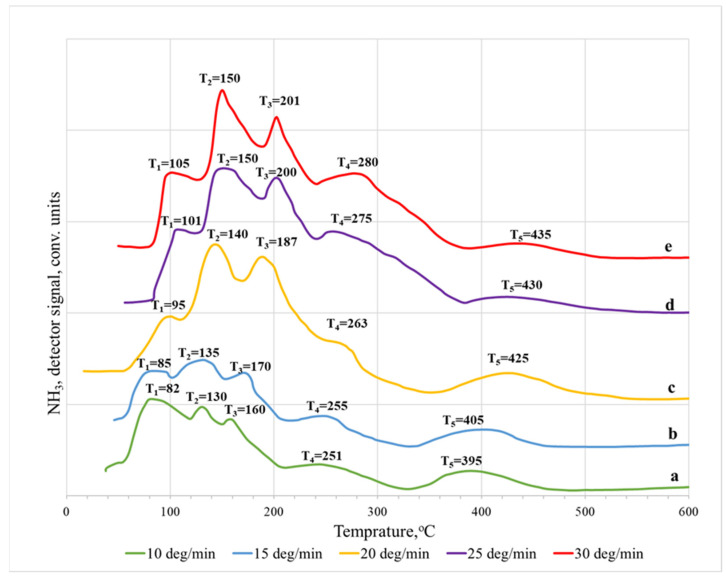
Thermal desorption spectra of NH_3_ on LTA membranes: (**a**) 10 °C/min; (**b**) 15 °C/min; (**c**) 20 °C/min; (**d**) 25 °C/min; (**e**) 30 °C/min.

The TPD spectra of ammonia desorbed from the surface of an LTA zeolite membrane (Figure 5) differ from the TPD spectra on a sodalite membrane and have five temperature maxima. T_1_, T_2_, and T_3_ also belong to the low-temperature region, which is characteristic of the desorption of physically adsorbed NH_3_ molecules [22]. Ammonia is adsorbed on acid sites, which most likely have electron-withdrawing properties, based on the structural features of LTA zeolite (Na_12_Al_2_Si_12_O_48_), which are tetrahedral compounds. Thus, desorption on the LTA membrane in the region of weak acid sites is more complex (multistage). The intensity of the high-temperature peak during desorption on the LTA membrane is higher than that of sodalite, but the peak in the region of medium acidity is, on the contrary, less intense. In addition, with an increase in the crystal size, the maximum of the high-temperature peak shifts from 360 to 400–430 °C. This is explained by the fact that, under the condition of possible desorption of ammonia after removal from the acid site, the position of the peak maximum is influenced by both the structural features of the pores and the total number of acid sites [18,19,21,22]. Therefore, with a more significant number of acid sites of the same type, the maximum of the corresponding peak shifts towards higher temperatures.

From the obtained TPD spectra recorded on both types of membranes, it is possible to draw some conclusions about the effect of the heating rate; namely, at a higher heating rate, 30 °C/min, the units of desorbed NH_3_ increases compared to 10 °C/min, since the system receives more energy, which allows for more efficient desorption. These results agree with the available literature data [23]. Moreover, at higher heating rates, the desorption maximum shifts towards a higher temperature, especially for an LTA membrane. This behavior is often associated with restrictions on mass transfer within particles [23].

**Figure 6:**
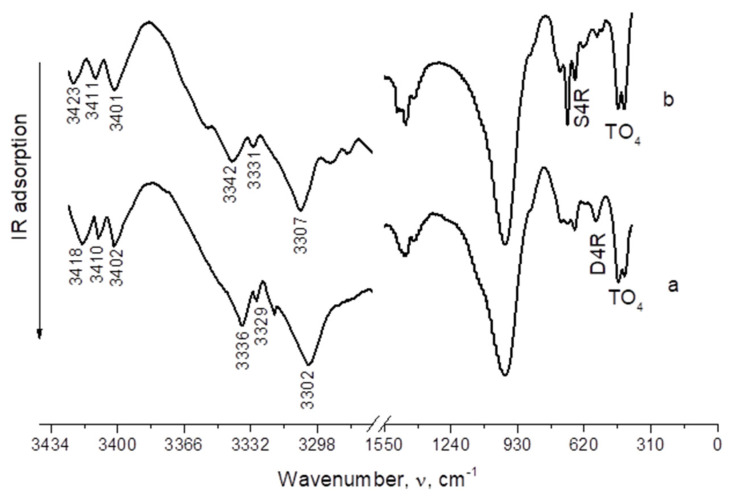
IR spectra of adsorbed ammonia on LTA (**a**) SOD; (**b**) zeolite membranes.

Investigation of the IR spectra of ammonia (Figure 6) adsorbed on the surface of the LTA and SOD zeolite membranes showed the presence of absorption bands in the range 3302–3307 and 3329–3331 cm^–1^, which were assigned to asymmetric valence and deformation vibrations of the adsorbed ammonia, respectively, which forms a coordination bond with the proton centers of the surface [24]. Weaker absorption bands were attributed to the ammonium ion remaining on the surface of the zeolites. Molecular adsorption of ammonia occurs during the specific interaction of ammonia molecules with the surface of the zeolite with the formation of a bond. The absorption bands of molecular adsorbed ammonia were observed at about 3401–3423 cm^–1^ on both types of membranes. The absorption band of ammonia molecules with a frequency of 3336 and 3342 cm^–1^ is characterized by a coordination bond with a Lewis center. These bands were interpreted as asymmetric valence deformation vibrations of an ammonium ion [25,26,27,28,29,30,31]. The absence of absorption bands at 3650 and 3680 cm^–1^ indicates that the corresponding hydroxyl groups are acid sites. Moreover, they confirm the presence of Lewis acid centers in the samples along with the Bronsted centers.

In this study, the acidity of each center was calculated, and the total acidity for both membranes was determined (Table 1 and Table 2). For the LTA zeolite membrane, its values are in the range of 0.08 × 10^19^ units/m^2^ while for the sodalite membrane, its value is substantially lower at 0.006 × 10^19^ units/m^2^. At the same time, the reliability of the results obtained is indicated by the fact that the total acidity does not depend on the heating rate of the samples; all the results are within the experimental error.

**Figure 7:**
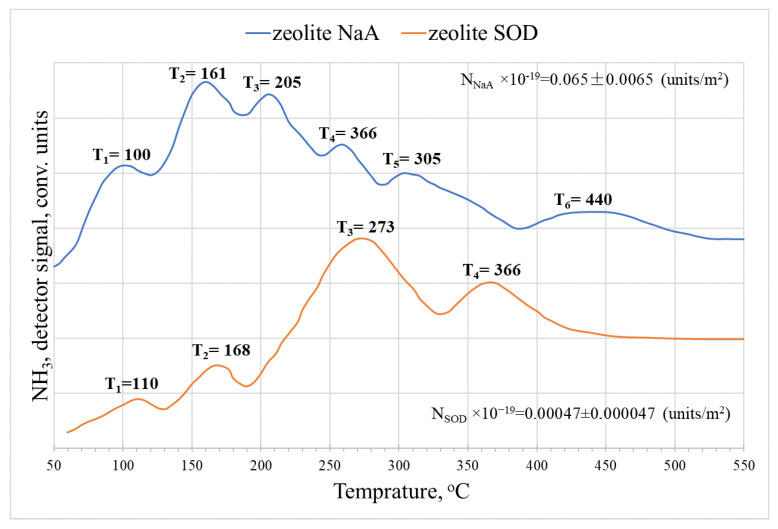
Thermal desorption spectra of NH_3_ on SOD and LTA zeolites, recorded at a heating rate of 30 °C/min.

The TPD spectra of ammonia for LTA and SOD zeolites (Figure 7) have the same number of temperature maxima, nature, and number of peaks, but the total number of acid sites is 20–25% less compared to zeolite membranes of the same type. Various authors have studied the kinetics of ammonia desorption on zeolites [32].

An essential characteristic determining the sorption and catalytic properties of zeolites in acid-base reactions is the binding energy of ammonia with the sample surface [25,26].

**Figure 8:**
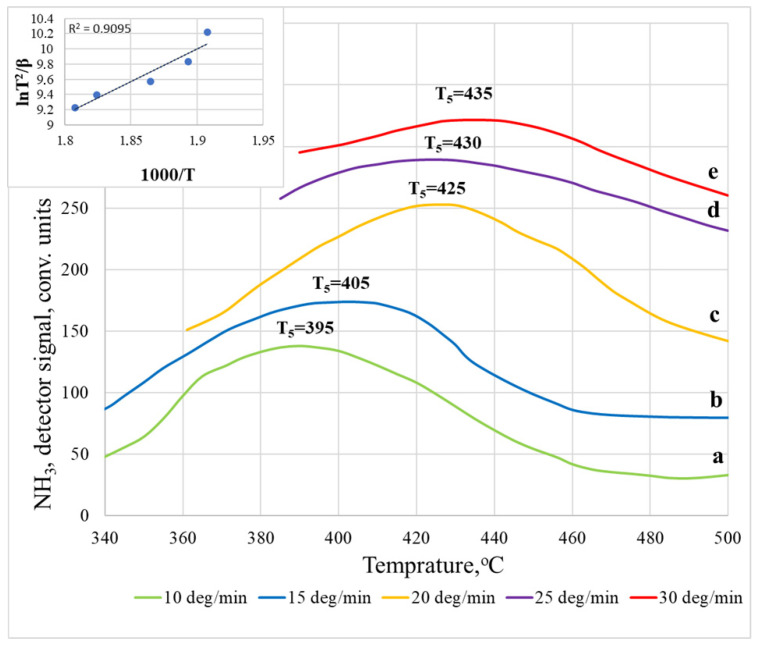
The results of studying the thermal desorption of ammonia on the LTA membrane at different heating rates. The inset in the left corner shows the temperature dependence (in Kissinger coordinates) of the maximum TPD peak on the heating rate.

**Figure 9:**
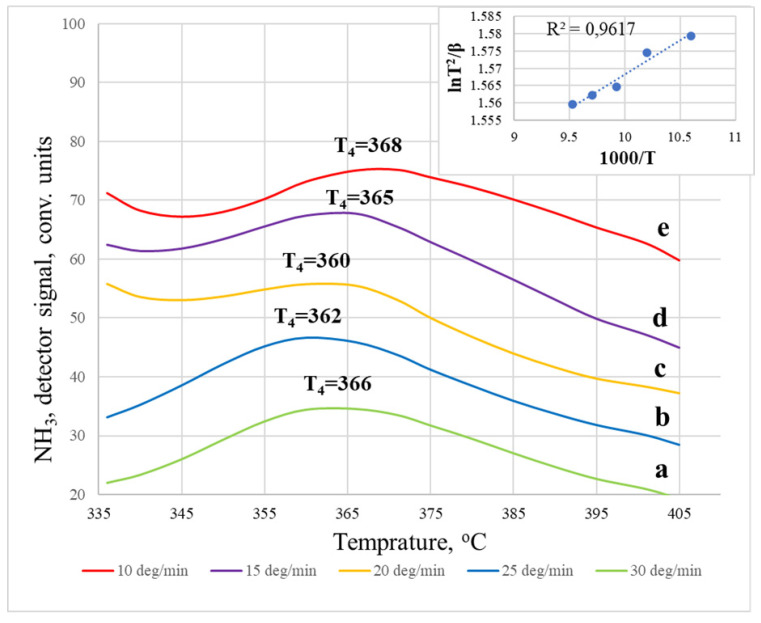
The results of studying the thermal desorption of ammonia on the SOD membrane at different heating rates. The inset in the left corner shows the temperature dependence (in Kissinger coordinates) of the maximum TPD peak on the heating rate.

Let us consider a more detailed calculation of the activation energy using the example of the most pronounced peak on the TPD curve for the LTA zeolite membrane (Figure 8) and the SOD membrane (Figure 9), the location of which depends on the removal rate in the temperature range of 350–500 °C. The temperature maxima for the LTA membrane, recorded at different heating rates of the spectra, are located as follows: T_1_ (10 °C/min) = 395 °C, T_2_ (15 °C/min) = 405 °C, T_3_ (20 °C/min) = 425 °C, T_4_ (25 °C/min) = 430 °C, and T_5_ (30 °C/min) = 435 °C. Using the Kissinger coordinates ($\ln\frac{T_{j}^{2}}{\beta }$ oт $\frac{1}{T_{j}}$), we obtained a linear dependence, and the tangent of the slope angle of a given curve allowed us to calculate the activation energy [27,28]. For this case, Ea = 52.18 ± 2.42 kJ/mol. The calculation of the high-temperature peak for the sodalite membrane, the activation energy of which is 49.94 ± 3.99 kJ/mol, was carried out similarly.

The data resulting from the calculation of the activation energies for all TPD curves of the SOD and LTA zeolite membrane are summarized in Table 3 and Table 4, respectively. It is worth noting that a directly proportional relationship between the strength of the acid sites and the activation energy values is observed [29]. For the LTA zeolite membrane in the temperature range of 50–200 °C, the activation energies are 20–35 kJ/mol (Table 4). This corresponds to the desorption of ammonia from α-cages. A further increase in the activation energy of the ammonia desorption to 70–80 kJ/mol is associated with the removal of ammonia from the less accessible β-cages of the LTA zeolite.

In contrast, the simpler and close-packed structure of the SOD membrane in the same temperature ranges shows lower values of the activation energy; namely, 10–20 kJ/mol for the processes of physical desorption of ammonia from the membrane surface, and 30–70 kJ/mol for the high-temperature regions (Table 3).

The data obtained are in good agreement with the fact that surface phenomena occur only when there is an excess of free energy in the boundary layer or in the presence of surface energy, which decreases proportionally to the surface area of the zeolite membrane [11,16,18]. Consequently, the qualitative characteristics of zeolite materials are directly proportional to changes in their surface area and structure [10,18]. Thus, the large specific surface area of the LTA membrane (150 m^2^/g) concerning the SOD membrane (115 m^2^/g), and the presence in the first case of the α and β cavities in the sample, providing access to OH groups, determines the increased content of Bronsted acid sites.

**Figure 10:**
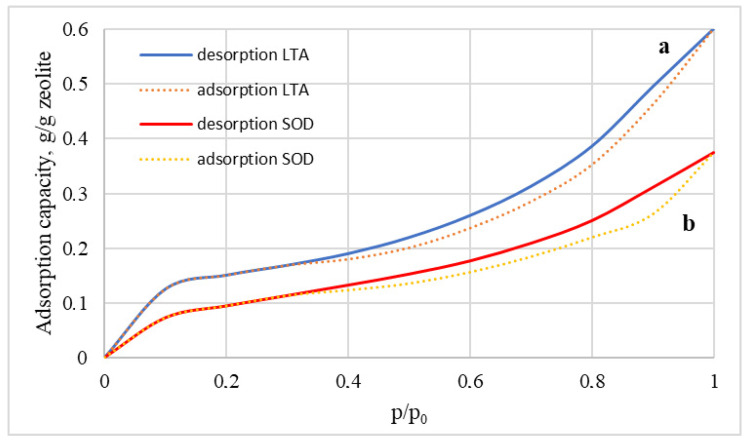
Adsorption and desorption curves of water vapor on LTA (**a**) SOD; (**b**) zeolite membranes.

If we link the data on the content of the acid-base groups with the properties of adsorption/desorption of water vapor on these types of membranes, then we can say that the isotherms display hysteresis and belong to type IV, which indicates the presence of capillary condensation in the zeolite membranes (Figure 10). The maximum moisture capacity of the LTA zeolite membrane is about 60 wt%, which is 1.5 times that of the SOD membrane. These facts are explained by the presence of SOD in zeolite only as β-cavities while LTA zeolite also has α-cavities with a higher adsorption capacity. Consequently, it is not the developed surface of the sorbent that is critical for the adsorption process but the presence of regular cavities in the zeolite framework. A similar pattern for LTA and SOD zeolites is also observed regarding the processes of adsorption/desorption of water, which was confirmed in a previous study, where it was shown that the increased characteristics of LTA zeolite compared to sodalite are associated with the presence of alpha and beta cells in the structure of the first one [33].

## 4. Conclusions

In the course of this study, a comparative analysis of the acidic properties of the surface of LTA and SOD zeolite membranes was carried out. The membranes were obtained using ultrasonic treatment by applying a zeolite layer on Al_2_O_3_ supports and subsequent hydrothermal crystallization of the precursors obtained on the substrate surface. As a result of synthesis, defect-free well-crystallized membranes were formed. The surface area and the shape of the “assembly” of sodalite cages are the factors determining the presence of Bronsted acid sites on the surface of the samples and consequently, their reactivity is due to the surface area and the shape of the “assembly” of sodalite cages. The increased content of acid sites on an LTA membrane is determined by the presence of α and β cages in the structure, which facilitates access to additional OH groups in the sample and contributes to increased sorption characteristics when studying the properties of membranes for adsorption/desorption of water vapor.

The study results were confirmed by processing kinetic data based on the use of the Kissinger model. The apparent activation energy values for the desorption of ammonia from zeolite membranes with LTA and SOD were calculated. It was found that at temperatures below 250 °C, the degree of conversion, the activation energy is no more than 35 kJ/mol, which corresponds to the desorption of physically bound ammonia. An increase in the activation values up to 70 kJ/mol (for SOD) and up to 80 kJ/mol (for LTA) is associated with the desorption of chemically bound ammonia from the samples.

## Figures and Tables

**Table 1:** The content of the acid sites on the surface of the SOD zeolite membrane, determined by the method of temperature-programmed desorption of NH_3_.

Heating Rate *β*, °C/min	Temperature Maximum, *T_i_*		Acidity Corresponding to *N_i_* × 10^−19^, Units/m^2^		Total Acidity, Corresponding to ⅀*N*_(*i*−*j*)_ × 10^−19^, Units/m^2^
10	T_1_	106	N_1_	0.00012 ± 0.000012	0.006 ± 0.0006
	T_2_	161	N_2_	0.00029 ± 0.000029	
	T_3_	270	N_3_	0.0033 ± 0.00033	
	T_4_	366	N_4_	0.00229 ± 0.000229	
15	T_1_	108	N_1_	0.0001 ± 0.00001	0.0065 ± 0.00065
	T_2_	163	N_2_	0.0003 ± 0.00003	
	T_3_	265	N_3_	0.0036 ± 0.00036	
	T_4_	362	N_4_	0.0024 ± 0.00024	
20	T_1_	107	*N_1_*	0.0001 ± 0.00001	0.0063 ± 0.00063
	T_2_	162	*N_2_*	0.0003 ± 0.00003	
	T_3_	261	*N_3_*	0.0035 ± 0.00035	
	T_4_	360	*N_4_*	0.0023 ± 0.00023	
25	T_1_	105	N_1_	0.0001 ± 0.00001	0.0059 ± 0.00059
	T_2_	161	N_2_	0.0003 ± 0.00003	
	T_3_	266	N_3_	0.0033 ± 0.00033	
	T_4_	365	N_4_	0.0022 ± 0.00022	
30	T_1_	108	N_1_	0.0001 ± 0.00001	0.0062 ± 0.00062
	T_2_	162	N_2_	0.0003 ± 0.00003	
	T_3_	275	N_3_	0.0035 ± 0.00035	
	T_4_	368	N_4_	0.0023 ± 0.00023	

**Table 2:** The content of acid sites on the surface of the LTA zeolite membrane, determined by the method of temperature-programmed desorption of NH_3_.

Heating Rate *β*, °C/min	Temperature Maximum, *T_i_*		Acidity Corresponding to *N_i_* × 10^−19^, Units/m^2^		Total Acidity, Corresponding to ⅀*N*_(*i*−*j*)_ × 10^−19^, Units/m^2^
10	T_1_	82	N_1_	0.0120 ± 0.00120	0.08 ± 0.008
	T_2_	130	N_2_	0.0210 ± 0.00210	
	T_3_	160	N_3_	0.0185 ± 0.00185	
	T_4_	251	N_4_	0.0155 ± 0.00155	
	T_5_	395	N_5_	0.013 ± 0.0013	
15	T_1_	85	N_1_	0.0121 ± 0.00121	0.079 ± 0.0079
	T_2_	135	N_2_	0.0394 ± 0.00394	
	T_3_	170	N_3_	0.0118 ± 0.00118	
	T_4_	255	N_4_	0.0058 ± 0.00058	
	T_5_	405	N_5_	0.0099 ± 0.00099	
20	T_1_	95	N_1_	0.0036 ± 0.00036	0.0773 ± 0.00773
	T_2_	140	N_2_	0.0333 ± 0.00333	
	T_3_	187	N_3_	0.0279 ± 0.00279	
	T_4_	263	N_4_	0.0027 ± 0.00027	
	T_5_	425	N_5_	0.0098 ± 0.00098	
25	T_1_	101	N_1_	0.0058 ± 0.00058	0.0801 ± 0.00801
	T_2_	150	N_2_	0.0312 ± 0.00312	
	T_3_	200	N_3_	0.0253 ± 0.00253	
	T_4_	275	N_4_	0.0081 ± 0.00081	
	T_5_	430	N_5_	0.0097 ± 0.00097	
30	T_1_	105	N_1_	0.0067 ± 0.00067	0.083 ± 0.0083
	T_2_	150	N_2_	0.0363 ± 0.00363	
	T_3_	201	N_3_	0.0235 ± 0.00235	
	T_4_	280	N_4_	0.0076 ± 0.00076	
	T_5_	435	N_5_	0.0089 ± 0.00089	

**Table 3:** Acidic properties of the SOD zeolite membrane obtained by NH_3_ desorption.

SOD Membrane Heating Rate, °C/min	Temperature Maximum	*T_max_*, °C	E_a_, kJ/mol
10	T_1_	106	12.74 ± 1.02
15		108	
20		107	
25		105	
30		108	
10	T_2_	161	18.04 ± 1.26
15		163	
20		162	
25		161	
30		162	
10	T_3_	270	32.3 ± 1.715
15		265	
20		261	
25		266	
30		275	
10	T_4_	366	69.94 ± 3.99
15		362	
20		360	
25		365	
30		368	

**Table 4:** Acidic properties of the LTA zeolite membrane obtained by NH_3_ desorption.

LTA Membrane Heating Rate, °C/min	Temperature Maximum	*T_max_*, °C	E_a_, kJ/mol
10	T_1_	82	20.24 ± 1.21
15		85	
20		95	
25		101	
30		105	
10	T_2_	130	28.31 ± 1.98
15		135	
20		140	
25		150	
30		150	
10	T_3_	160	35.72 ± 1.88
15		170	
20		187	
25		200	
30		201	
10	T_4_	251	74.26 ± 4.91
15		255	
20		263	
25		275	
30		280	
10	T_5_	395	78.18 ± 2.42
15		405	
20		425	
25		430	
30		435	

## Data Availability

Not applicable.
